# Motivation and Perceived Barriers to Physical Activity Among Lithuanian Students: A Cross-Sectional Study

**DOI:** 10.3390/medicina62081556

**Published:** 2026-08-13

**Authors:** Dominykas Radišauskas, Aušra Beržanskytė

**Affiliations:** Institute of Health Sciences, Faculty of Medicine, Vilnius University, LT-03101 Vilnius, Lithuania; ausra.berzanskyte@mf.vu.lt

**Keywords:** physical activity, prevalence, motivation, barriers, factors, habits, students

## Abstract

*Background and Objectives*: Regular physical activity is important for students’ health and well-being, but participation may be influenced by motivation and perceived barriers. This study assessed physical activity levels, motivational regulation, perceived barriers, and their associations among students enrolled in Lithuanian higher education institutions. *Materials and Methods*: A cross-sectional survey was conducted among 370 students. The questionnaire assessed weekly physical activity, motivational regulation, perceived barriers, and sociodemographic characteristics. Group comparisons and exploratory hierarchical multivariable logistic regression were performed. *Results*: Overall, 74.6% of the participants met the World Health Organization physical activity recommendations. In separate analyses, male participants and those from major cities were more likely to meet the World Health Organization physical activity recommendations. Intrinsic and identified regulation were the most prominent motivational factors, whereas lack of energy, lack of willpower, and lack of time were the most frequently reported barriers. In the final model, higher intrinsic regulation was associated with greater odds of meeting the recommendations (odds ratio 1.64, 95% confidence interval 1.10–2.44), while lack of willpower (odds ratio 0.34, 95% confidence interval 0.19–0.60) and lack of time (odds ratio 0.35, 95% confidence interval 0.18–0.68) were associated with lower odds. Sociodemographic characteristics were not statistically significant in the final model. *Conclusions*: Most participants in this sample met the World Health Organization physical activity recommendations. The findings highlight the relevance of intrinsic regulation and selected perceived barriers to physical activity participation. These exploratory associations may help guide future longitudinal and intervention research on context-specific physical activity promotion in higher education.

## 1. Introduction

Physical activity (PA) is an essential part of everyday life that has a positive impact on human health. It is well established that adequate PA improves functional capacity, reduces the risk of disease, excess weight, depression, and anxiety, while also enhancing overall mood, and emotional well-being [1]. In addition, PA is associated with better mental health, increased longevity, and greater resistance to non-communicable and chronic diseases [2]. These associations were first identified more than 70 years ago in a study of London transport workers, which found that physically active bus conductors had nearly half the risk of death from coronary heart disease compared to less physically active bus drivers [2].

It can be said that safe and responsible engagement in PA plays a major role in promoting public health and serves as an effective preventive strategy for maintaining and improving both physical and mental well-being among individuals and society as a whole [3]. However, despite the widespread belief that PA is the foundation of good health, this attitude is often difficult to implement in practice. According to a 2024 World Health Organization (WHO) fact sheet, 31% of adults worldwide did not meet the recommended levels of PA, while more than 80% of adolescents were insufficiently physically active [4]. It is also important to note that insufficient PA imposes a significant economic burden on public health systems. It is estimated that if no action is taken to increase PA levels between 2020 and 2030, up to 500 million new cases of chronic non-communicable diseases could occur worldwide, with treatment costs reaching approximately 300 billion U.S. dollars over this period, which corresponds to around 27 billion U.S. dollars annually [5].

Insufficient PA is also common among students, who, due to this new stage of life, often experience a more sedentary lifestyle. Such a lifestyle may have negative health consequences, including overweight, various types of cancer, cardiovascular diseases, diabetes, and increased mortality [6]. In addition, a sedentary and passive lifestyle, which is often associated with prolonged use of digital technologies such as computers and smartphones, is negatively related to mental health among young people [7]. Optimal mental health is an important component of a high-quality and fulfilling life, as it enables individuals to feel well, function effectively in daily activities, and maintain harmonious relationships with their environment. In children and adolescents, PA can serve as an effective means of addressing psychological difficulties such as depression, stress, and negative emotional experiences [7]. Regarding students, it has been shown that PA can significantly reduce symptoms of depression, anxiety, and stress [8].

A large international study involving 23 countries found that only 58.6% of students met the WHO PA recommendations, with regional prevalence ranging from 49.4% to 67.6% [9]. In a study conducted in Ireland, 64.3% of students met the WHO PA recommendations, while 27.5% were insufficiently active and 8.1% reported no PA at all [10]. In Spanish universities, 77.6% of students met the WHO PA recommendations. However, only 44.4% did so when considering leisure-time PA alone [11]. In a study conducted in Saudi Arabia, 69.9% of students engaged in regular PA, although a significant proportion of activity was related to occupational or daily tasks [12]. Overall, the findings from the literature indicate that in different countries a substantial proportion of students do not achieve the recommended levels of PA.

The implementation of measures aimed at promoting PA is of significant importance. However, for their successful implementation among the younger generation, it is necessary to identify the main factors shaping PA habits, the motivations for engaging in PA, and, most importantly, the barriers that prevent young people from doing so. A proper understanding of these aspects helps to develop effective intervention programmes and health policies aimed at promoting healthy behaviour [13]. It is important to emphasize that fundamental habits are formed at a young age and have a lasting impact on quality of life both during adolescence and in later adulthood. Therefore, the integration of PA in early life is an essential and integral factor ensuring good physical and mental health.

Although factors related to students’ PA have been widely studied, research often focuses on PA prevalence or separate sociodemographic correlates [1,3,14]. In Lithuania, studies related to PA have also been conducted. However, the primary focus has been on monitoring and assessing PA levels in the population [15,16]. Fewer studies provide an integrated perspective that considers PA levels, motivational regulation, and perceived barriers within the same student sample, particularly in the Lithuanian higher education context. Motivation and barriers represent related but distinct components of PA behaviour: motivational regulation reflects the reasons and degree of autonomy underlying participation, whereas perceived barriers capture practical, social, and personal constraints that may hinder participation even when motivation is present. Considering these dimensions together may therefore provide a more comprehensive understanding of the discrepancy between students’ willingness to be physically active and their actual participation. This integrated approach extends beyond studies focusing only on PA prevalence or on either motivation or barriers separately by allowing motivational resources and perceived constraints to be examined within the same sample. Accordingly, the present study aimed to assess PA levels, motivational regulation, and perceived barriers among the surveyed students enrolled in Lithuanian higher education institutions, examine their associations with sociodemographic characteristics, and explore whether sociodemographic characteristics, motivational regulation, and perceived barriers were independently associated with meeting the WHO PA recommendations.

## 2. Materials and Methods

### 2.1. Ethical Principles

The study was conducted in accordance with the Declaration of Helsinki and established ethical principles ensuring participants’ privacy, autonomy, and anonymity, and was approved by the Research Ethics Committee of the Faculty of Medicine at Vilnius University on 5 February 2025 (Approval No. (1.7 E) 150000-KT-50).

All participants were informed about the aims of the study, the research procedures, and their rights as research participants. Participation in the study was entirely voluntary, and respondents were free to withdraw from or decline participation at any stage without any consequences.

All collected data were fully anonymous, and the database contained no personally identifiable information.

### 2.2. Sample and Organization of the Study

Participants were recruited using a non-probability convenience sampling method. Eligible participants were students enrolled at universities or colleges in Lithuania who had sufficient proficiency in Lithuanian to understand and complete the questionnaire, as the survey was administered exclusively in Lithuanian. Consequently, international and exchange students without sufficient Lithuanian-language proficiency were not eligible to participate.

Data were collected using an anonymous online questionnaire developed in Google Forms (Google LLC, Mountain View, CA, USA; https://workspace.google.com/products/forms/). The questionnaire was disseminated through social media platforms and the Vilnius University Faculty of Medicine website. Participation was voluntary. As the survey was distributed through open online channels and the number of students who received or viewed the invitation was unknown, a response rate could not be calculated.

The sample size was estimated using the Raosoft Sample Size Calculator (Raosoft, Inc., Seattle, WA, USA; https://raosoftcalculator.net/), assuming a 95% confidence level, a 5% margin of error, and a 50% response distribution. According to the State Data Agency, 102.3 thousand students were enrolled in Lithuanian universities and colleges at the beginning of the 2022–2023 academic year [17]. Based on this population estimate, the calculated sample size was 383 respondents. As a non-probability sampling method was used, this calculation was treated as a sample-size benchmark and did not imply that the sample was nationally representative.

The survey was conducted between 7 February and 16 March 2025. A total of 388 questionnaires were received. After incomplete or incorrectly completed questionnaires were excluded, 370 responses were included in the final statistical analysis, which was slightly below the calculated sample-size benchmark.

### 2.3. Data Collection Instrument and Measures

The survey instrument (Appendix A) was assembled for the purposes of the present study and consisted of four sections: sociodemographic questions, two study-specific questions assessing PA, and Lithuanian-language versions of two instruments assessing motivational regulation and perceived barriers.

The first section collected sociodemographic information about the respondents, including gender, age, place of residence, and study- and work-related circumstances. This section enabled the characterization of the study sample and facilitated the analysis of factors associated with students’ PA habits from different perspectives.

The second section did not include the complete WHO Global Physical Activity Questionnaire (GPAQ). Instead, it comprised two study-specific PA questions developed with reference to the GPAQ descriptions of moderate- and high-intensity PA [18]. Each question first asked whether the participant engaged in the respective intensity of PA during the week and, if so, requested the total duration in minutes per week. Examples of activities were provided to facilitate interpretation. Unlike the complete GPAQ, these questions did not assess PA separately across occupational, transport, and recreational domains or assess sedentary behaviour. They were therefore used only to estimate weekly moderate- and high-intensity PA and to classify participants according to the WHO PA recommendations. Accordingly, the psychometric properties reported for the complete GPAQ cannot be assumed to apply to these modified study-specific questions. The classification of participants according to PA level was based on the WHO PA recommendations for adults aged 18–64 years [19]. Participants were classified as meeting the WHO PA recommendations if they accumulated at least 150 min of moderate-intensity PA, at least 75 min of high-intensity PA, or an equivalent combination of the two per week. The average weekly time spent in PA was calculated in minutes. Total PA was estimated by combining moderate- and high-intensity activities. To account for differences in activity intensity, high-intensity PA was converted into moderate-intensity equivalents using the following formula: 1 min of high-intensity activity = 2 min of moderate-intensity activity. The converted high-intensity activity was then added to moderate-intensity activity to obtain the total weekly PA expressed in moderate-intensity-equivalent minutes. Accordingly, participants accumulating at least 150 moderate-intensity-equivalent minutes per week were classified as meeting the WHO PA recommendations, whereas those accumulating less than 150 min were classified as not meeting it.

The third section used the Behavioural Regulation in Exercise Questionnaire-2 (BREQ-2), which is publicly available and widely used and validated in research examining motivation for PA [20,21,22]. The BREQ-2 consists of 19 items and assesses behavioural regulation in exercise across five categories, reflecting different types of motivational regulation. Each item is rated on a five-point Likert scale ranging from 0 to 4, where 0 indicates “strongly disagree” and 4 indicates “strongly agree.” The results are summarised by calculating the mean score for each category (Table 1).

The fourth section of the questionnaire consisted of the Barriers to Being Active (BBA) instrument designed to identify barriers to PA. This questionnaire is also freely available online and has been validated and widely used in research examining factors that may be associated with PA [23,24]. The BBA questionnaire consists of 21 items, which allow the identification of the main barriers limiting PA. It is structured into seven categories, each containing three items that specifically represent a particular barrier domain. Each item is rated on a four-point Likert scale ranging from 0 to 3, where 0 indicates “very unlikely” and 3 indicates “very likely.” The scores for each category are summed, and a total score of 5 or more indicates that a given barrier is considered a significant factor limiting PA (Table 2).

The BREQ-2 and BBA items were translated into Lithuanian for the present study. The translation did not follow a complete published forward–backward translation or comprehensive cross-cultural adaptation protocol. Before the main data collection, the complete online questionnaire—including the sociodemographic items, the two study-specific PA questions developed with reference to the GPAQ descriptions, and the Lithuanian-language BREQ-2 and BBA items—was pilot-tested with five students. The pilot test was used to assess the technical administration of the survey, completeness of responses, and data coding and scoring procedures. All five participants completed all questionnaire items, and no major technical or coding problems were identified. Given the small pilot sample, this procedure was regarded as a preliminary feasibility assessment rather than formal psychometric or cross-cultural validation of the questionnaire or its individual components.

Internal consistency was assessed using Cronbach’s α for both the complete instruments and their individual subscales. Cronbach’s α was 0.83 for the complete BREQ-2 and 0.89 for the complete BBA. At the subscale level, Cronbach’s α ranged from 0.578 to 0.943 for the BREQ-2 and from 0.535 to 0.842 for the BBA (Table 1 and Table 2). Lower internal consistency was observed for the amotivation, social influence, and lack of resources subscales. Therefore, findings involving these subscales were interpreted with caution. The relatively small number of items within individual subscales, particularly the three-item BBA subscales, should also be considered when interpreting the coefficients.

### 2.4. Statistical Analysis

The dataset was created, checked, and cleaned in Microsoft Excel 2019 (version 2508, Build 19127.20302; Microsoft Corporation, Redmond, WA, USA). Statistical analyses were performed using R (version 4.3.3; R Foundation for Statistical Computing, Vienna, Austria) through the R Commander interface. Categorical variables were summarised using frequencies and percentages, whereas continuous variables were described using means. The distributions of continuous variables were assessed by visual inspection of histograms, comparison of means and medians, skewness and kurtosis, and the Shapiro–Wilk test. Most continuous variables were considered sufficiently close to a normal distribution for parametric analyses. Moderate-intensity, high-intensity, and total PA (minutes/week) showed positively skewed distributions and were additionally described using medians and interquartile ranges (IQR). Means were retained for descriptive purposes and to facilitate comparisons with previous studies.

Statistical tests were selected according to variable type and distribution. Associations between categorical variables were examined using the chi-square (χ^2^) test. Differences in moderate-intensity, high-intensity, and total PA between male and female participants were analysed using the Mann–Whitney U test because these variables were not normally distributed. Comparisons involving more than two groups were performed using one-way analysis of variance (ANOVA) followed by Tukey’s post hoc test when the assumption of homogeneity of variance was met. When Levene’s test indicated unequal variances, Welch’s ANOVA followed by Games–Howell post hoc comparisons was applied. Effect sizes were reported alongside *p*-values and expressed as Cramér’s V for chi-square tests, rank-biserial correlation for Mann–Whitney U tests, eta-squared (η^2^) for omnibus analyses of variance, and Hedges’ g for pairwise post hoc comparisons. Statistical significance was set at α = 0.05, and 95% confidence intervals (CI) were calculated where appropriate.

Two distinct sets of binary logistic regression analyses were performed. First, separate barrier-specific binary logistic regression models examined associations between sociodemographic characteristics and individual perceived barriers. Reporting the respective barrier was specified as the modelled event. Sociodemographic variables significantly associated with each barrier in bivariate chi-square analyses (*p* < 0.05) were entered into the corresponding multivariable model. Gender was included in the models for lack of time, social influence, lack of energy, and lack of skills; place of residence was additionally included in the social influence model; and age group was included in the lack-of-energy and lack-of-resources models. Employment status and type of higher education institution were not included because they were not significantly associated with any barrier. Lack of willpower and fear of injury were not modelled because no sociodemographic variable showed a significant bivariate association with these barriers.

Second, an exploratory hierarchical binary logistic regression analysis comprising three nested models was conducted to examine factors associated with meeting the WHO PA recommendations. Meeting the WHO PA recommendations was specified as the modelled event. Model 1 included sociodemographic characteristics (gender, age group, place of residence, employment status, and type of higher education institution). Model 2 additionally included intrinsic and identified regulation as indicators of autonomous motivation. Model 3 further included the three most frequently reported barriers (lack of energy, lack of willpower, and lack of time). Predictor selection was based on conceptual relevance, prevalence, and model parsimony rather than on bivariate statistical significance.

Odds ratios (OR) with 95% confidence intervals were reported for all logistic regression models, and Wald tests were used to assess the statistical significance of individual predictors. Overall model fit was evaluated using likelihood-ratio (LR) tests and McFadden’s pseudo-R^2^, whereas model discrimination was assessed using the area under the receiver operating characteristic curve (ROC AUC). Nested hierarchical models were compared using LR tests, and multicollinearity was assessed using variance inflation factors. Sensitivity, specificity, overall classification accuracy, balanced accuracy, and majority-class accuracy were calculated using a predicted probability threshold of 0.50. Because of the imbalance between outcome categories, overall classification accuracy was interpreted together with majority-class accuracy rather than in isolation.

## 3. Results

### 3.1. Participant Characteristics

The mean age of the participants was 21 years, and most (86.5%; *n* = 320) were aged 18–23 years. Females accounted for 75.7% (*n* = 280) of the sample. Before entering higher education, participants had most commonly lived in cities or towns (43.8%; *n* = 162) or major cities (43.5%; *n* = 161). Most respondents were university students (88.4%; *n* = 327), and just over half were employed during their studies (53.5%; *n* = 198) (Table 3).

### 3.2. Prevalence of PA Among Students

Overall, 74.6% (95% CI: 69.9–78.8) of participants met the WHO PA recommendations. Statistically significant differences were observed according to gender and place of residence. Male participants and students from major cities were more likely to meet the WHO PA recommendations than female participants and those from smaller towns or rural areas, although the corresponding effect sizes were small (Table 4). No statistically significant differences were found according to age, type of higher education institution, or employment status.

**Figure 1:**
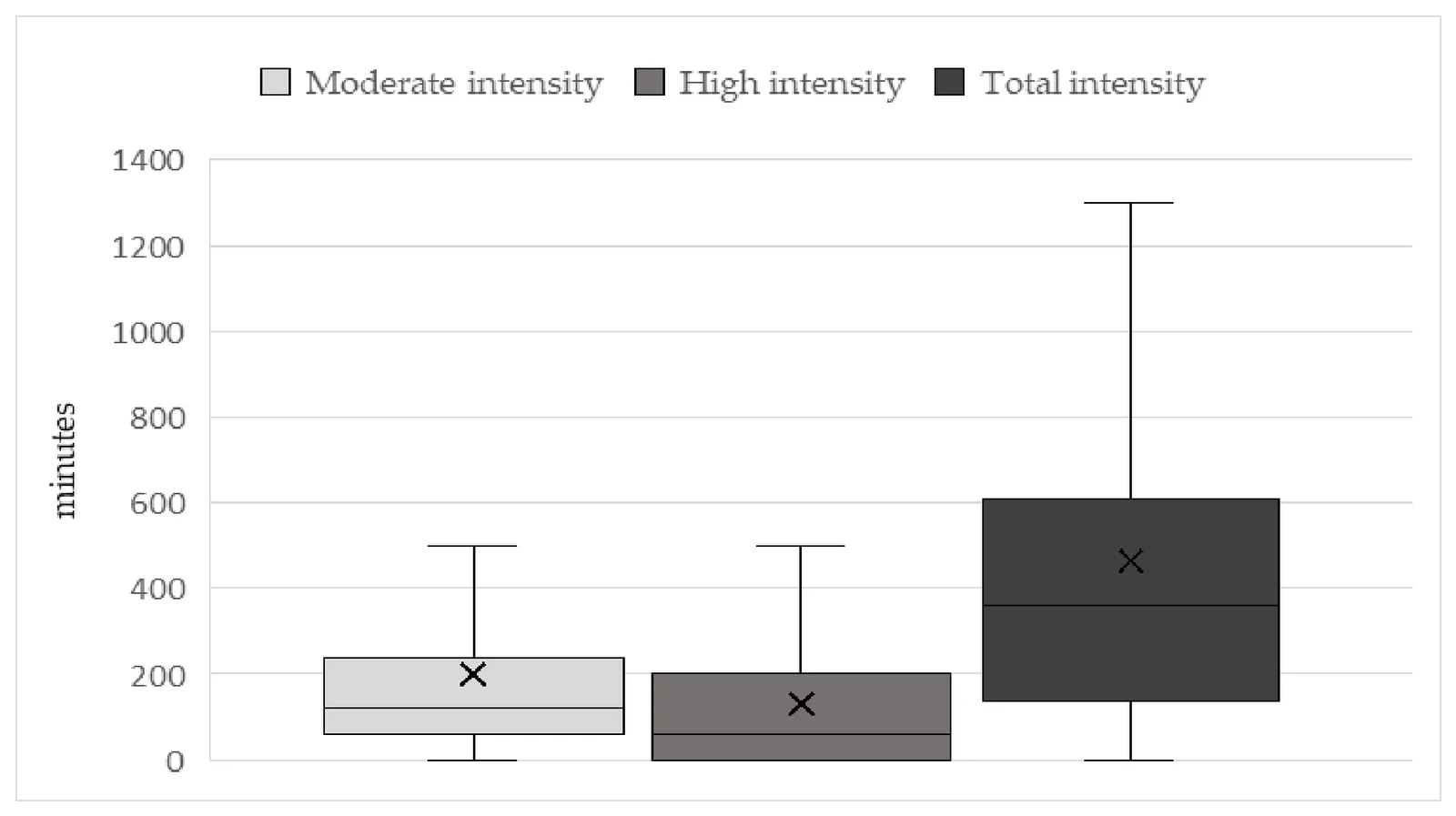
Students’ PA per week (minutes) by intensity.

Participants accumulated an average of 200.7 min/week of moderate-intensity PA and 131.3 min/week of high-intensity PA, corresponding to a mean total moderate-equivalent PA of 463.3 min/week. As expected from the positively skewed distributions, median values were consistently lower than the corresponding means (Figure 1).

**Figure 2:**
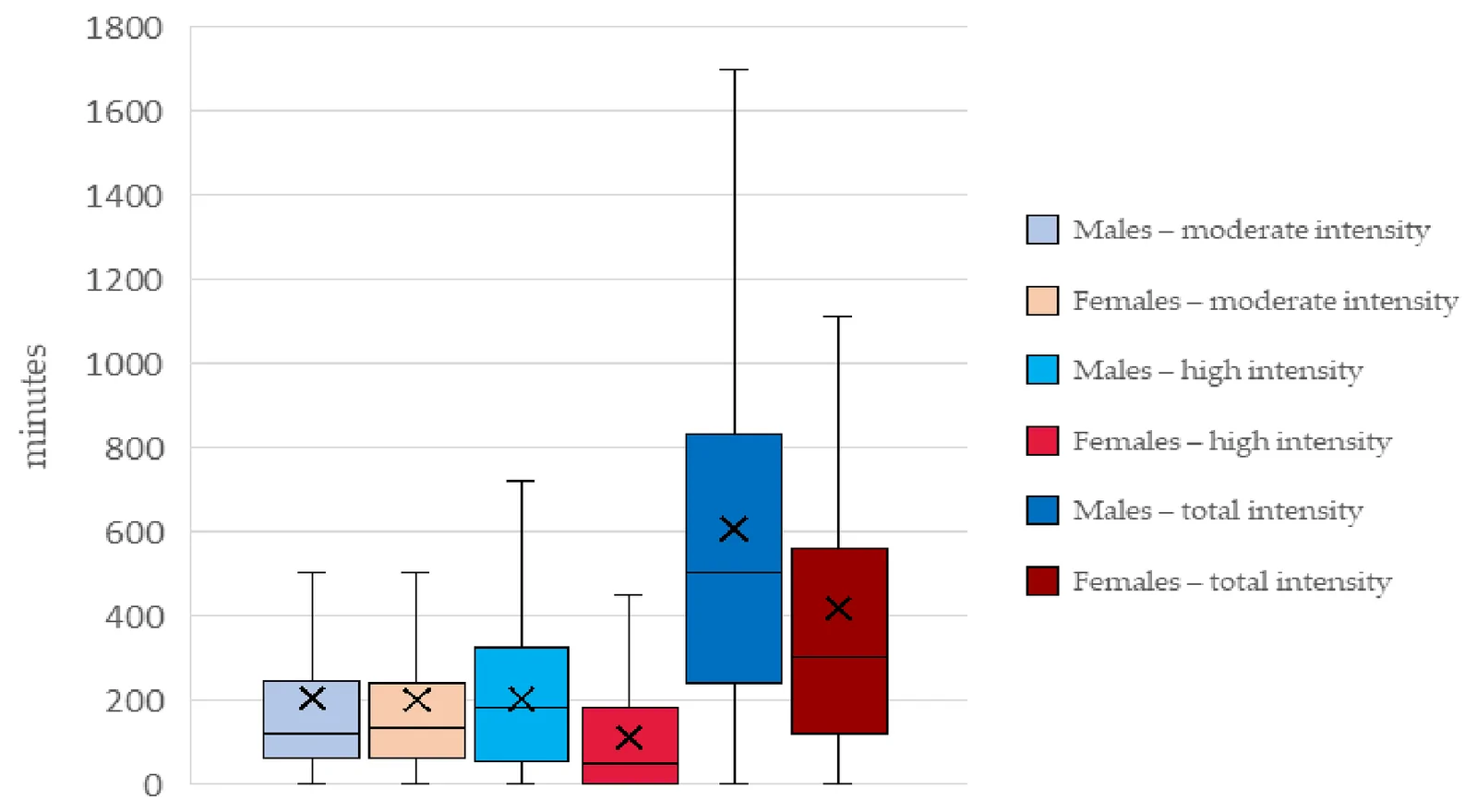
Distribution of weekly PA duration according to gender.

Moderate-intensity PA was similar between males and females. The mean weekly duration was 203.4 min among males and 199.8 min among females, while the corresponding medians were 120 min (IQR: 60–240) and 132.5 min (IQR: 60–240), respectively. The difference was not statistically significant and the effect size was negligible (Mann–Whitney U = 12,349.5, *p* = 0.776, rank-biserial correlation = −0.020). High-intensity PA was higher among males, with a mean of 201.2 min/week and a median of 180 min/week (IQR: 60–315), compared with a mean of 108.8 min/week and a median of 47.5 min/week (IQR: 0–180) among females. This difference was statistically significant and of moderate magnitude (U = 16,781.5, *p* < 0.001, rank-biserial correlation = 0.332). Total PA was also higher among males: the mean was 605.8 min/week and the median was 500 min/week (IQR: 255–825), compared with 417.4 min/week and 300 min/week (IQR: 120–560), respectively, among females. The difference was statistically significant and the effect size was small (U = 16,088.0, *p* < 0.001, rank-biserial correlation = 0.277) (Figure 2).

### 3.3. Motivational Factors Associated with Students’ PA

Intrinsic regulation had the highest mean score (2.74), followed by identified regulation (2.57). Mean scores were lower for introjected regulation (1.69) and external regulation (0.42), whereas amotivation showed the lowest value (0.27) (Table 5). Findings concerning amotivation should be interpreted cautiously because this subscale demonstrated lower internal consistency (Cronbach’s α = 0.578).

Motivational regulation varied according to place of residence. Students from major cities consistently showed the highest mean scores for intrinsic, identified, introjected, and external regulation, whereas students from rural areas showed the lowest scores. These differences were statistically significant, although the corresponding effect sizes were small. In contrast, amotivation did not differ statistically significantly between the groups (Table 6). Pairwise post hoc comparisons and corresponding Hedges’ g effect sizes are presented in Table 6.

### 3.4. Barriers to Students’ PA

The most frequently reported barriers to PA were lack of energy (39.5%), followed by lack of willpower (33.8%) and lack of time (33.0%). Lack of resources (20.0%), social influence (18.4%), lack of skills (7.6%), and fear of injury (2.7%) were reported less frequently (Table 7). Findings concerning social influence and lack of resources should be interpreted cautiously because these subscales demonstrated lower internal consistency (Cronbach’s α = 0.598 and 0.535, respectively).

Binary logistic regression analysis revealed that females had approximately twice the odds of reporting lack of time as a significant barrier to PA compared with males (OR = 2.0). In addition, females had 3.2 times higher odds of reporting social influence as a barrier, 2.9 times higher odds of reporting lack of energy, and 4.5 times higher odds of reporting lack of skills than males.

Regarding age groups, students aged 18–23 years had 3.2 times higher odds of reporting lack of resources than students aged 24–29 years. Students from rural areas had 2.6 times higher odds than those from major cities of reporting social influence as a significant barrier to PA. Although students living in cities or towns also had higher odds of reporting this barrier than those from major cities (OR = 1.3), the association was not statistically significant (Table 8).

McFadden’s pseudo-R^2^ values ranged from 0.014 to 0.046, and ROC AUC values ranged from 0.551 to 0.641 (Table 8). At the predicted probability threshold of 0.50, all five models classified participants into the majority “barrier not reported” category (Table 9). These findings indicate limited discrimination between students who did and did not report the respective barriers.

### 3.5. Factors Associated with Meeting the WHO PA Recommendations

An exploratory hierarchical multivariable logistic regression analysis was conducted to examine factors associated with meeting the WHO PA recommendations. In Model 1, females had 50% lower odds of meeting the recommendation than males (OR = 0.50). Participants aged 18–23 years had 2.1 times higher odds of meeting the recommendation than those aged 24–29 years (OR = 2.11). Compared with participants living in major cities, those living in cities or towns had 44% lower odds (OR = 0.56), while those living in rural areas had 56% lower odds of meeting the recommendation (OR = 0.44). Type of higher education institution and employment status were not significantly associated with meeting the recommendation (Table 10).

After intrinsic and identified regulation were added in Model 2, intrinsic regulation was significantly associated with meeting the recommendation. A one-point increase in intrinsic regulation was associated with 2.2 times higher odds of meeting the recommendation (OR = 2.15). Identified regulation was not statistically significant. The associations with gender, age group, and place of residence observed in Model 1 were attenuated and were no longer statistically significant after motivational regulation was included.

In Model 3, which additionally included lack of energy, lack of willpower, and lack of time, intrinsic regulation remained significantly associated with meeting the recommendation (OR = 1.64). Participants reporting lack of willpower had 66% lower odds of meeting the recommendation than those who did not report this barrier (OR = 0.34). Similarly, participants reporting lack of time had 65% lower odds of meeting the recommendation (OR = 0.35). Identified regulation, lack of energy, and the remaining sociodemographic characteristics were not statistically significant in the final model (Table 10). LR testing showed that adding motivational regulation significantly improved model fit compared with Model 1 (LR χ^2^ = 62.16, *p* < 0.001). Adding perceived barriers further improved model fit compared with Model 2 (LR χ^2^ = 23.75, *p* < 0.001).

Overall model fit and discriminatory performance improved across the three models. McFadden’s pseudo-R^2^ increased from 0.038 in Model 1 to 0.186 in Model 2 and 0.243 in Model 3. Likewise, ROC AUC increased from 0.634 to 0.787 and 0.825, respectively (Table 10). No evidence of problematic multicollinearity was observed, as all variance inflation factors were ≤3.04.

At the predicted probability threshold of 0.50, Model 1 classified nearly all participants as meeting the WHO PA recommendations, whereas classification performance improved after motivational regulation and perceived barriers were added (Table 11). The final model achieved the highest overall classification performance but remained more accurate in identifying participants who met the recommendation than those who did not.

## 4. Discussion

The results of the present study revealed that 74.6% of participants met the WHO PA recommendations. Similar findings have been reported in studies conducted in other countries, such as Spain (77.6%) and the United States (76%), where compliance with the WHO PA recommendations was slightly higher, whereas in Ireland this proportion was lower, reaching 64.3% [10,11,25]. An even less favourable situation was observed in countries from Africa, Asia, and South America, where WHO PA recommendations were met by only 58.6% of participants overall [9]. However, these cross-study comparisons should be interpreted cautiously because the studies differed in their sampling strategies, participant characteristics, PA assessment methods, and definitions or domains of PA included in the analyses.

Although the prevalence of sufficient PA observed in the present study was relatively favourable in an international context, findings from previous Lithuanian studies have been highly inconsistent. One study reported that 91.6% of students met the WHO PA recommendations. However, the majority of participants were from Vilnius University and health sciences programmes [26]. Other studies have reported substantially lower or highly variable compliance rates, ranging from 11.9% and 51.3% to 94.5% and 100% [27,28,29,30]. This variability may reflect differences in study periods, including the COVID-19 pandemic, as well as differences in study populations, which often consisted of students from only one or two higher education institutions or programmes closely related to PA.

Lower PA levels among female participants were consistent with findings from previous studies [9,10,11,26,28]. The difference was most pronounced for high-intensity PA, for which the effect size was moderate, whereas the difference in total PA was small and that in moderate-intensity PA was negligible.

The proportion of participants meeting the WHO PA recommendations was higher among those from major cities than among those from rural areas. Because access to sports facilities, socioeconomic circumstances, occupational or household PA, and differences in how PA was perceived and reported were not assessed, explanations involving urban–rural differences in these factors should be regarded as hypotheses for future research rather than conclusions supported directly by the present data.

Intrinsic and identified regulation were the most prominent forms of motivation. This pattern is consistent with studies conducted in other countries, which similarly identified enjoyment and perceived health benefits as prominent motives for PA participation among university students [31,32]. Direct comparison of motivation scores should nevertheless be made cautiously because of differences in cultural context, participant characteristics, recruitment procedures, and questionnaire administration.

From the perspective of Self-Determination Theory, the higher scores for intrinsic and identified regulation indicate that autonomous forms of motivation were more prominent than controlled forms of motivation. Intrinsic regulation reflects participation because the activity itself is enjoyable, whereas identified regulation reflects engagement because PA is personally valued and considered beneficial. The relatively low scores for external regulation and amotivation suggest that external pressure and a lack of perceived purpose were less prominent reasons for PA participation in this sample [21,33].

The hierarchical multivariable analysis further showed that intrinsic regulation remained associated with meeting the WHO PA recommendations after sociodemographic characteristics and perceived barriers were considered, whereas identified regulation did not remain statistically significant. This finding may indicate that enjoyment of PA had a more independent association with participation than recognising its health-related value alone. Given the cross-sectional design, the direction of this association cannot be established: intrinsic motivation may support PA participation, but greater participation may also strengthen enjoyment of PA.

Intrinsic and identified regulation were higher among participants from major cities than among those from rural areas. The corresponding effect sizes were small, indicating that place of residence accounted for only limited differences in motivational regulation. These findings should therefore be interpreted as modest group-level associations rather than evidence of substantial motivational differences between residential groups.

Lack of energy, lack of willpower, and lack of time were the most frequently reported barriers, consistent with previous research among student populations [34,35,36]. Lack of resources and social influence were less frequent, while lack of skills and fear of injury were reported least often. Previous studies suggest that study-related fatigue, financial costs, access to exercise opportunities, social support, embarrassment, and discomfort in public exercise settings may contribute to these barriers [34,37,38,39]. Because these contextual and psychosocial factors were not directly assessed, they should be regarded as possible explanations for future research rather than mechanisms demonstrated by the present study.

Female participants had higher odds of reporting lack of energy, social influence, and lack of skills, whereas younger participants had higher odds of reporting lack of resources. These findings identify group differences in reported barriers but do not establish the mechanisms underlying them. Self-efficacy, confidence, concerns about social evaluation, financial independence, parental support, and the stability of daily routines were not measured and should be examined in future studies.

Although several sociodemographic characteristics were significantly associated with specific perceived barriers, the overall fit and discriminatory performance of the logistic regression models were limited. McFadden’s pseudo-R^2^ values ranged from 0.014 to 0.046, indicating only limited improvement in model fit compared with the corresponding intercept-only models. Similarly, the ROC AUC values (0.551–0.641) indicated only modest discrimination. Therefore, the statistically significant odds ratios should be interpreted as group-level associations rather than evidence of strong individual-level predictive ability. These findings suggest that sociodemographic characteristics alone are insufficient to distinguish reliably between students who do and do not report particular barriers.

The exploratory hierarchical analysis extended the separate group comparisons by considering sociodemographic characteristics, motivational regulation, and perceived barriers within the same model. Adding motivational regulation and perceived barriers improved model fit and discrimination compared with the sociodemographic model alone. Nevertheless, the findings should be interpreted as exploratory associations rather than causal effects.

Lack of willpower and lack of time were independently associated with lower odds of meeting the WHO PA recommendations. In contrast, lack of energy, although the most frequently reported barrier, was not statistically significant after the other variables were considered. This indicates that the frequency of a reported barrier does not necessarily correspond to the strength of its independent association with meeting the WHO PA recommendations.

The associations of gender, age, and place of residence observed in the sociodemographic model were no longer statistically significant after motivation and barriers were included. This attenuation indicates overlap between these groups of variables but should not be interpreted as evidence of mediation. Although the final model showed relatively good apparent discrimination according to the ROC AUC, its specificity remained limited and its performance was not internally or externally validated. Therefore, it should not be interpreted as a tool for individual prediction.

Several limitations should be acknowledged. First, the cross-sectional design does not allow causal relationships to be inferred. Second, participants were recruited using non-probability convenience sampling through social media and the Vilnius University Faculty of Medicine website. Because the survey was distributed through open online channels and the number of students who received the invitation was unknown, the response rate could not be calculated. Consequently, selection bias due to voluntary participation and non-response bias cannot be excluded.

Females accounted for 75.7% of the sample, resulting in a substantial gender imbalance. Students who were more interested in health, PA, or survey participation may also have been more likely to respond. In addition, dissemination through the Vilnius University Faculty of Medicine website may have resulted in an overrepresentation of health sciences students. Because participants were not classified according to their institution, study programme, or academic field, the extent of this potential overrepresentation could not be quantified.

The final analytic sample of 370 respondents was also slightly below the planned recruitment target. These sampling characteristics limit the generalisability of the findings and the results should not be interpreted as national prevalence estimates for all Lithuanian students. Instead, they describe the associations and patterns observed among the students who participated in the survey.

PA, motivation, and barriers were assessed using self-reported data, which may be affected by recall and social desirability bias. Because both perceived barriers and compliance with the WHO PA recommendations were derived from self-reported responses, some conceptual and measurement overlap and common-method bias cannot be excluded. Barriers such as lack of time, lack of energy, and lack of willpower may partly reflect participants’ current PA behaviour in addition to perceived constraints, which may have influenced the magnitude or direction of the associations observed in Model 3, including the non-significant positive estimate for lack of energy. Some forms of everyday PA, including household tasks, active commuting, gardening, or physical labour, may not have been fully captured or consistently recognised by respondents. The Lithuanian-language BREQ-2 and BBA items and the two study-specific PA questions developed with reference to the GPAQ were not subjected to a complete published forward–backward translation, cross-cultural adaptation, and psychometric validation protocol. Although the complete questionnaire was pilot-tested with five students, this small pilot primarily assessed feasibility, response completeness, and data coding and scoring procedures and did not establish the validity of the adapted measures. Consequently, measurement error and limited comparability with studies using the original validated instruments cannot be ruled out. Further validation of these measures in Lithuanian student samples is needed.

Because numerous statistical tests were performed without a global adjustment for multiple comparisons, an increased risk of type I error cannot be excluded. Findings with *p*-values close to the significance threshold should therefore be interpreted cautiously. Some subscales, particularly amotivation, social influence, and lack of resources, demonstrated lower internal consistency, and findings related to these constructs should also be interpreted cautiously. In addition, some estimates, particularly those involving low-frequency barriers and sparse subgroup counts, were accompanied by wide CI, limiting their precision and requiring cautious interpretation. In the barrier-specific logistic regression models, sociodemographic predictors were selected based on statistical significance in the bivariate analyses. This data-driven approach may have excluded potentially relevant confounders, retained chance associations, and increased the dependence of the models on the present sample; therefore, these models should be regarded as exploratory. The hierarchical multivariable analysis was exploratory and was developed and evaluated within the same sample without internal or external validation. Therefore, its discrimination and classification performance may be optimistic and may not generalise to other student populations. The lower number of participants who did not meet the WHO PA recommendations may also have contributed to the limited specificity. Future studies should use probability-based or stratified recruitment, aim for a more balanced gender distribution, collect detailed information on institutions, study programmes, and academic fields.

Nevertheless, the study had several strengths. A notable strength was the use of a structured instrument that simultaneously assessed motivational regulation and perceived barriers to PA. This integrative approach provided a more comprehensive understanding of students’ PA behaviour by capturing both determinants of engagement and constraints limiting participation. To our knowledge, relatively few studies in Lithuania have examined these dimensions simultaneously. The findings may inform future research and support the development of more targeted PA promotion strategies in higher education settings.

## 5. Conclusions

The findings of the present study indicate that most surveyed students met the WHO PA recommendations, while lower PA levels were observed among female participants and those from rural areas in the separate analyses. These patterns may warrant further investigation when developing and evaluating PA promotion initiatives. The integration of accessible PA opportunities into study programmes could be explored as one potential approach, but its effectiveness should be assessed in future intervention studies.

Participants generally recognised the value and benefits of PA, with enjoyment and perceived health benefits emerging as the most prominent motivational factors. Higher intrinsic regulation was also associated with greater odds of meeting the WHO PA recommendations in the exploratory multivariable analysis. These findings suggest that enjoyment and the perceived value of PA may be relevant considerations for future PA promotion initiatives; their practical effectiveness should be evaluated in intervention studies.

Lack of energy, lack of willpower, and lack of time were the most frequently reported barriers. In the exploratory multivariable analysis, lack of willpower and lack of time were associated with lower odds of meeting the WHO PA recommendations, whereas lack of energy was not independently associated with the outcome. Although the inclusion of motivational regulation and perceived barriers improved model performance, the findings should be interpreted as exploratory group-level associations rather than as evidence of causal relationships or as a sufficient basis for individual prediction.

Overall, the findings may help guide future longitudinal and intervention studies aimed at developing and evaluating context-specific PA promotion strategies in higher education. Further studies with more representative samples are needed to confirm these associations before firm recommendations can be made.

## Figures and Tables

**Table 1:** BREQ-2 questionnaire structure and description of subscales.

Categories (Regulatory Types)	Statement Describing the Category (Regulatory Type)	Item Numbers	Cronbach’s α
Amotivation	“I do not see the point in engaging in physical activity”	5, 9, 12, 19	0.578
External regulation	“I engage in physical activity because other people say I should or because I feel pressure from others”	1, 6, 11, 16	0.818
Introjected regulation	“I feel bad when I do not engage in physical activity”	2, 7, 13	0.690
Identified regulation	“I value the benefits of physical activity”	3, 8, 14, 17	0.750
Intrinsic regulation	“I enjoy engaging in physical activity”	4, 10, 15, 18	0.943

**Table 2:** BBA questionnaire structure.

Categories (Barriers)	Item Numbers	Cronbach’s α
Lack of time	1, 8, 15	0.773
Social influence	2, 9, 16	0.598
Lack of energy	3, 10, 17	0.788
Lack of willpower	4, 11, 18	0.842
Fear of injury	5, 12, 19	0.676
Lack of skill	6, 13, 20	0.641
Lack of resources	7, 14, 21	0.535

**Table 3:** Characteristics of university and college students according to demographic and social factors.

	University Students *n* = 327 (88.4%)		College Students *n* = 43 (11.6%)		Total *n* = 370	
	*n*	%	*n*	%	*n*	%
Gender						
Males	78	23.9	12	27.9	90	24.3
Females	249	76.1	31	72.1	280	75.7
Place of residence (before studies)						
Major city	147	44.9	14	32.6	161	43.5
City or town	139	42.5	23	53.5	162	43.8
Rural area	41	12.6	6	13.9	47	12.7
Employment status (during studies)						
Employed	176	53.8	22	51.2	198	53.5
Unemployed	151	46.2	21	48.8	172	46.5
Age						
18–23 years	280	85.6	40	93.0	320	86.5
24–29 years	47	14.4	3	7.0	50	13.5

**Table 4:** Compliance with WHO PA recommendations according to social and demographic characteristics.

	Met Recommendations % (*n*)	95% CI	χ^2^ Test *p*-Value	Cramér’s V
Gender				
Males *n* = 90	83.3 (75)	74.3; 89.6	0.029	0.114
Females *n* = 280	71.8 (201)	66.2; 76.7		
Age				
18–23 years *n* = 320	75.9 (243)	71.0; 80.3	0.133	0.078
24–29 years *n* = 50	66.0 (33)	52.2; 77.6		
Higher education institution				
College *n* = 43	76.7 (33)	62.3; 86.8	0.731	0.018
University *n* = 327	74.3 (243)	69.3; 78.7		
Place of residence				
Major city *n* = 161	80.7 (130)	74.0; 86.1	0.033	0.136
City or town *n* = 162	71.6 (116)	64.2; 78.0		
Rural area *n* = 47	63.8 (30)	49.5; 76.0		
Employment status (during studies)			0.429	0.041
Employed *n* = 198	76.3 (151)	69.9; 81.7		
Unemployed *n* = 172	72.7 (125)	65.6; 78.8		

**Table 5:** Student motivation for PA.

Motivation (Regulatory Type)	Mean	95% CI
Intrinsic	2.74	2.63; 2.86
Identified	2.57	2.48; 2.66
Introjected	1.69	1.59; 1.79
External	0.42	0.35; 0.49
Amotivation	0.27	0.22; 0.32

**Table 6:** Student motivation for PA (by place of residence).

Motivation (Regulatory Type)	Major City Mean (95% CI) *n* = 161	Cities or Towns Mean (95% CI) *n* = 162	Rural Area Mean (95% CI) *n* = 47	F Test *p*-Value	Eta-Squared (η^2^)	Post Hoc Comparisons (*p*-Value; Hedges’ g)
Intrinsic	2.93 (2.77; 3.10)	2.70 (2.52; 2.88)	2.23 (1.89; 2.58)	<0.001	0.039	MC vs. CT (*p* = 0.147; g = 0.21); MC vs. RA (*p* < 0.001; g = 0.65); CT vs. RA (*p* = 0.031; g = 0.40).
Identified	2.78 (2.65; 2.90)	2.48 (2.34; 2.62)	2.18 (1.93; 2.42)	<0.001	0.055	MC vs. CT (*p* = 0.005; g = 0.35); MC vs. RA (*p* < 0.001; g = 0.74); CT vs. RA (*p* = 0.087; g = 0.34).
Introjected	1.88 (1.73; 2.03)	1.62 (1.45; 1.79)	1.28 (1.05; 1.52)	< 0.001 *	0.036	MC vs. CT (*p* = 0.075; g = 0.24); MC vs. RA (*p* < 0.001; g = 0.63); CT vs. RA (*p* = 0.055; g = 0.32).
External	0.51 (0.38; 0.63)	0.38 (0.29; 0.47)	0.23 (0.12; 0.34)	0.004 *	0.019	MC vs. CT (*p* = 0.239; g = 0.18); MC vs. RA (*p* = 0.003; g = 0.38); CT vs. RA (*p* = 0.089; g = 0.28).
Amotivation	0.29 (0.20; 0.38)	0.26 (0.19; 0.32)	0.22 (0.08; 0.35)	0.636	0.002	MC vs. CT (*p* = 0.801; g = 0.07); MC vs. RA (*p* = 0.652; g = 0.13); CT vs. RA (*p* = 0.892; g = 0.09).

MC —major city; CT—city or town; RA—rural area. * Levene’s test was statistically significant; therefore, Welch’s ANOVA and Games–Howell post hoc comparisons were used for introjected and external regulation. Tukey’s post hoc test was used for the remaining regulatory types.

**Table 7:** Main barriers limiting PA among students.

Barriers	Is This an Important Barrier Limiting PA? (%)	
	Yes (%)	95% CI
Lack of time	33.0	28.4; 37.9
Social influence	18.4	14.8; 22.6
Lack of energy	39.5	34.6; 44.5
Lack of willpower	33.8	29.2; 38.7
Fear of injury	2.7	1.5; 4.9
Lack of skills	7.6	5.3; 10.7
Lack of resources	20.0	16.2; 24.4

**Table 8:** Associations between sociodemographic characteristics and perceived barriers to PA.

	OR	(95% CI)	Wald *p*-Value	LR Test and *p*-Value	McFadden R^2^	ROC AUC (95% CI)
Independent variable	Dependent variable					
	Lack of time					
Females (ref. males)	2.0	(1.2; 3.6)	0.014	(6.53) *p* = 0.011	0.014	0.559 (0.516; 0.602)
	Social influence					
Females (ref. males)	3.2	(1.5; 7.8)	0.006	(16.14) *p* = 0.001	0.046	0.641 (0.573; 0.709)
Cities or towns (ref. major city)	1.3	(0.7; 2.4)	0.359			
Rural area (ref. major city)	2.6	(1.2; 5.6)	0.014			
	Lack of energy					
Females (ref. males)	2.9	(1.7; 5.0)	<0.001	(15.66) *p* < 0.001	0.032	0.588 (0.546–0.629)
18–23 years (ref. 24–29 years)	0.9	(0.5; 1.7)	0.713			
	Lack of skills					
Females (ref. males)	4.5	(1.3; 28.3)	0.043	(6.10) *p* = 0.016	0.031	0.593 (0.539; 0.647)
	Lack of resources					
18–23 years (ref. 24–29 years)	3.2	(1.3; 11.0)	0.030	(6.22) *p* = 0.013	0.017	0.551 (0.518; 0.584)

**Table 9:** Classification performance of the barrier-specific binary logistic regression models at a predicted probability threshold of 0.50.

Dependent Variable	Majority-Class Accuracy (%)	Model Accuracy (%)	Sensitivity (%)	Specificity (%)	Balanced Accuracy (%)
Lack of time	67.0	67.0	0.0	100.0	50.0
Social influence	81.6	81.6	0.0	100.0	50.0
Lack of energy	60.5	60.5	0.0	100.0	50.0
Lack of skills	92.4	92.4	0.0	100.0	50.0
Lack of resources	80.0	80.0	0.0	100.0	50.0

**Table 10:** Exploratory hierarchical multivariable logistic regression analysis of factors associated with meeting the WHO PA recommendations.

Dependent Variable	Model	Independent Variable	OR	95% CI	Wald *p*-Value	Overall LR Test vs. Intercept-Only Model and *p*-Value	McFadden R^2^	ROC AUC (95% CI)
Meeting the WHO PA recommendations	Model 1	Females (ref. males)	0.50	(0.27; 0.94)	0.032	(15.89) *p* = 0.014	0.038	0.634 (0.570; 0.698)
		18–23 years (ref. 24–29 years)	2.11	(1.05; 4.23)	0.036			
		College (ref. university)	1.14	(0.53; 2.46)	0.734			
		City or town (ref. major city)	0.56	(0.33; 0.97)	0.037			
		Rural area (ref. major city)	0.44	(0.21; 0.90)	0.026			
		Employed (ref. not employed)	1.30	(0.79; 2.15)	0.306			
Meeting the WHO PA recommendations	Model 2	Females (ref. males)	0.53	(0.26; 1.06)	0.072	(78.04) *p* < 0.001	0.186	0.787 (0.736; 0.838)
		18–23 years (ref. 24–29 years)	1.85	(0.85; 4.03)	0.122			
		College (ref. university)	1.35	(0.59; 3.12)	0.477			
		City or town (ref. major city)	0.72	(0.40; 1.30)	0.275			
		Rural area (ref. major city)	0.79	(0.35; 1.78)	0.576			
		Employed (ref. not employed)	1.14	(0.65; 1.99)	0.652			
		Intrinsic regulation (per one-point increase)	2.15	(1.48; 3.11)	<0.001			
		Identified regulation (per one-point increase)	1.27	(0.79; 2.04)	0.330			
Meeting the WHO PA recommendations	Model 3	Females (ref. males)	0.57	(0.27; 1.18)	0.128	(101.80) *p* < 0.001	0.243	0.825 (0.777; 0.872)
		18–23 years (ref. 24–29 years)	1.81	(0.79; 4.13)	0.160			
		College (ref. university)	1.22	(0.52; 2.86)	0.643			
		City or town (ref. major city)	0.78	(0.42; 1.45)	0.436			
		Rural area (ref. major city)	0.81	(0.35; 1.90)	0.632			
		Employed (ref. not employed)	1.15	(0.64; 2.08)	0.632			
		Intrinsic regulation (per one-point increase)	1.64	(1.10; 2.44)	0.016			
		Identified regulation (per one-point increase)	1.47	(0.89; 2.45)	0.133			
		Lack of energy: reported (ref. not reported)	1.56	(0.78; 3.13)	0.206			
		Lack of willpower: reported (ref. not reported)	0.34	(0.19; 0.60)	<0.001			
		Lack of time: reported (ref. not reported)	0.35	(0.18; 0.68)	0.002			

The LR tests reported in Table 10 compare each model with the corresponding intercept-only model. The incremental LR tests reported in the Results compare Model 2 with Model 1 and Model 3 with Model 2.

**Table 11:** Classification performance of the hierarchical binary logistic regression models for meeting the WHO PA recommendations at a predicted probability threshold of 0.50.

Dependent Variable	Model	Majority-Class Accuracy (%)	Model Accuracy (%)	Sensitivity (%)	Specificity (%)	Balanced Accuracy (%)
Meeting the WHO PA recommendations	Model 1	74.6	75.1	99.6	3.2	51.4
	Model 2	74.6	81.1	94.9	40.4	67.7
	Model 3	74.6	81.4	93.8	44.7	69.3

## Data Availability

The data presented in this study are available on request from the corresponding author. The data is not publicly available due to ethical restrictions.

## Appendix A

Questionnaire

Sociodemographic Questions

1. What is your gender:

☐ Male

☐ Female

2. What is your age? (please specify):

☐ ....... years

3. Which type of higher education institution do you study at?

☐ University

☐ College

4. Where did you live before starting your current studies?

☐ Large city (Vilnius, Kaunas, Klaipėda, Šiauliai, Panevėžys)

☐ Town or small city

☐ Rural area

5. Do you work (have an employment contract with an employer) during your studies?

☐ Yes

☐ No

Questions Assessing Physical Activity Prevalence

1. Do you engage in vigorous-intensity physical activity during the week that causes a substantial increase in breathing or heart rate (e.g., football, basketball, fitness training, weightlifting, aerobics, intensive running, etc.)?

☐ Yes

☐ No

1.1. If yes, how much time (in minutes) per week do you spend engaging in this type of physical activity?

☐ ... min

2. Do you engage in moderate-intensity physical activity during the week that causes a slight increase in breathing or heart rate (e.g., swimming, volleyball, light running, brisk walking, light household chores, cycling, etc.)?

☐ Yes

☐ No

2.1. If yes, how much time (in minutes) per week do you spend engaging in this type of physical activity?

☐ ... min

Questions Assessing Factors Influencing Physical Activity

BREQ-2 Questionnaire (Behavioural Regulation in Exercise)

Why do you engage in physical activity? (Please read each statement below and choose the answer that best reflects your opinion: 0—strongly disagree, 1–3—partially agree, 4—strongly agree.)

**No****.**	**Statement**	**Strongly Disagree**	**Partially Agree**			**Strongly Agree**
1.	I engage in physical activity because other people say I should	0 ☐	1 ☐	2 ☐	3 ☐	4 ☐
2.	I feel guilty when I do not engage in physical activity	0 ☐	1 ☐	2 ☐	3 ☐	4 ☐
3.	I value the benefits of physical activity	0 ☐	1 ☐	2 ☐	3 ☐	4 ☐
4.	I enjoy engaging in physical activity	0 ☐	1 ☐	2 ☐	3 ☐	4 ☐
5.	I do not see the point of engaging in physical activity	0 ☐	1 ☐	2 ☐	3 ☐	4 ☐
6.	I engage in physical activity because my friends/parents/partner say I should	0 ☐	1 ☐	2 ☐	3 ☐	4 ☐
7.	I feel bad when I miss a workout	0 ☐	1 ☐	2 ☐	3 ☐	4 ☐
8.	It is important for me to engage in physical activity regularly	0 ☐	1 ☐	2 ☐	3 ☐	4 ☐
9.	I do not understand why physical activity should matter to me	0 ☐	1 ☐	2 ☐	3 ☐	4 ☐
10.	I enjoy my physical activity sessions	0 ☐	1 ☐	2 ☐	3 ☐	4 ☐
11.	I engage in physical activity because others would be disappointed in me if I did not	0 ☐	1 ☐	2 ☐	3 ☐	4 ☐
12.	I do not see why I should engage in physical activity	0 ☐	1 ☐	2 ☐	3 ☐	4 ☐
13.	I feel like a failure when I cannot engage in physical activity for some time	0 ☐	1 ?	2 ☐	3 ?	4 ☐
14.	I think it is important to make the effort to engage in physical activity regularly	0 ☐	1 ☐	2 ☐	3 ☐	4 ☐
15.	I consider physical activity to be an enjoyable activity	0 ?	1 ☐	2 ☐	3 ☐	4 ☐
16.	I feel pressure from my friends/parents to engage in physical activity	0 ☐	1 ☐	2 ☐	3 ☐	4 ☐
17.	I feel restless if I cannot engage in physical activity regularly	0 ☐	1 ☐	2 ☐	3 ☐	4 ?
18.	Participating in physical activity gives me pleasure and satisfaction	0 ☐	1 ☐	2 ☐	3 ☐	4 ?
19.	I think physical activity is a waste of time	0 ☐	1 ☐	2 ?	3 ☐	4 ☐

Questions Assessing External Barriers Negatively Affecting Physical Activity

BBA Questionnaire (Barriers to Being Active)

What prevents you from being physically active? (Below are reasons people give to describe why they do not engage in as much physical activity as they think they should. Please read each statement and indicate how likely you are to say each of them.)

**How****Likely Are You to Say?**	**Very Unlikely**	**Somewhat Unlikely**	**Somewhat Likely**	**Very Likely**
1. My day is so busy now, I just don’t think I can make the time to include physical activity in my regular schedule	0 ☐	1 ☐	2 ☐	3 ☐
2. None of my family members or friends like to do anything active, so I don’t have a chance to exercise	0 ☐	1 ☐	2 ☐	3 ☐
3. I’m just too tired after work to get any exercise	0 ☐	1 ☐	2 ☐	3 ☐
4. I’ve been thinking about getting more exercise, but I just can’t seem to get started	0 ☐	1 ☐	2 ☐	3 ☐
5. I’m getting older so exercise can be risky	0 ☐	1 ☐	2 ☐	3 ☐
6. I don’t get enough exercise because I have never learned the skills for any sport	0 ☐	1 ☐	2 ☐	3 ☐
7. I don’t have access to jogging trails, swimming pools, bike paths, etc.	0 ☐	1 ☐	2 ☐	3 ☐
8. Physical activity takes too much time away from other commitments—time, work, family, etc.	0 ☐	1 ☐	2 ☐	3 ☐
9. I’m embarrassed about how I will look when I exercise with others	0 ☐	1 ☐	2 ☐	3 ☐
10. I don’t get enough sleep as it is. I just couldn’t get up early or stay up late to get some exercise	0 ☐	1 ☐	2 ☐	3 ☐
11. It’s easier for me to find excuses not to exercise than to go out to do something	0 ☐	1 ☐	2 ☐	3 ☐
12. I know of too many people who have hurt themselves by overdoing it with exercise	0 ☐	1 ☐	2 ☐	3 ☐
13. I really can’t see learning a new sport at my age	0 ☐	1 ☐	2 ☐	3 ☐
14. It’s just too expensive. You have to take a class or join a club or buy the right equipment	0 ☐	1 ☐	2 ☐	3 ☐
15. My free times during the day are too short to include exercise	0 ☐	1 ☐	2 ☐	3 ☐
16. My usual social activities with family or friends do not include physical activity	0 ☐	1 ☐	2 ☐	3 ☐
17. I’m too tired during the week and I need the weekend to catch up on my rest	0 ☐	1 ☐	2 ☐	3 ☐
18. I want to get more exercise, but I just can’t seem to make myself stick to anything	0 ☐	1 ☐	2 ☐	3 ☐
19. I’m afraid I might injure myself or have a heart attack	0 ☐	1 ☐	2 ☐	3 ☐
20. I’m not good enough at any physical activity to make it fun	0 ☐	1 ☐	2 ☐	3 ☐
21. If we had exercise facilities and showers at work, then I would be more likely to exercise	0 ☐	1 ☐	2 ☐	3 ☐
